# Detection of BDNF-Related Proteins in Human Perilymph in Patients With Hearing Loss

**DOI:** 10.3389/fnins.2019.00214

**Published:** 2019-03-26

**Authors:** Ines de Vries, Heike Schmitt, Thomas Lenarz, Nils Prenzler, Sameer Alvi, Hinrich Staecker, Martin Durisin, Athanasia Warnecke

**Affiliations:** ^1^Department of Otolaryngology, Hannover Medical School, Hanover, Germany; ^2^Cluster of Excellence Hearing4all, German Research Foundation, Hannover Medical School, Hanover, Germany; ^3^Department of Otolaryngology, Head and Neck Surgery, University of Kansas School of Medicine, Kansas City, MO, United States

**Keywords:** inner ear, perilymph, BDNF, neurotrophin, bioinformatic analysis, proteomics, diagnostics, cochlear implant

## Abstract

The outcome of cochlear implantation depends on multiple variables including the underlying health of the cochlea. Brain derived neurotrophic factor (BDNF) has been shown to support spiral ganglion neurons and to improve implant function in animal models. Whether endogenous BDNF or BDNF-regulated proteins can be used as biomarkers to predict cochlear health and implant outcome has not been investigated yet. Gene expression of BDNF and downstream signaling molecules were identified in tissue of human cochleae obtained from normal hearing patients (*n* = 3) during skull base surgeries. Based on the gene expression data, bioinformatic analysis was utilized to predict the regulation of proteins by BDNF. The presence of proteins corresponding to these genes was investigated in perilymph (*n* = 41) obtained from hearing-impaired patients (*n* = 38) during cochlear implantation or skull base surgery for removal of vestibular schwannoma by nanoscale liquid chromatography coupled to tandem mass spectrometry (nano LC-MS/MS). Analyzed by mass spectrometry were 41 perilymph samples despite three patients undergoing bilateral cochlear implantation. These particular BDNF regulated proteins were not detectable in any of the perilymph samples. Subsequently, targeted analysis of the perilymph proteome data with Ingenuity Pathway Analysis (IPA) identified further proteins in human perilymph that could be regulated by BDNF. These BDNF regulated proteins were correlated to the presence of residual hearing (RH) prior to implantation and to the performance data with the cochlear implant after 1 year. There was overall a decreased level of expression of BDNF-regulated proteins in profoundly hearing-impaired patients compared to patients with some RH. Phospholipid transfer protein was positively correlated to the preoperative hearing level of the patients. Our data show that combination of gene expression arrays and bioinformatic analysis can aid in the prediction of downstream signaling proteins related to the BDNF pathway. Proteomic analysis of perilymph may help to identify the presence or absence of these molecules in the diseased organ. The impact of such prediction algorithms on diagnosis and treatment needs to be established in further studies.

## Introduction

One of the challenges in cochlear implantation is to understand the wide variability in performance amongst users. Candidates with similar history and implanted with the same device can demonstrate outcomes on both ends of the spectrum (Carlson et al., 2012). A plethora of factors may contribute to the variance in speech perception including age, duration of deafness, genetics, surgical technique and device characteristics, neuronal survival, electrode position, or central processing abilities (Carlson et al., 2012; Causon et al., 2015; Kral et al., 2016; Shearer et al., 2017). Since human temporal bone studies have recently shown a link between spiral ganglion population and cochlear implant outcomes (Seyyedi et al., 2014), one of the research targets is to maintain the viable population of spiral ganglion neurons for stimulation. Objective measures of cochlear function also suggest that a healthier neuronal population may result in better speech outcomes (Kim et al., 2010). This raises the possibility of modulating spiral ganglion function and health through drug delivery to the inner ear in conjunction with cochlear implantation.

Neurotrophins are the most promising candidates to support the auditory nerve by increasing neuronal survival. Within the murine inner ear, BDNF and NT-3 seem to regulate the connection of hair cells and neurons (Pirvola et al., 1992, 1997; Flores-Otero and Davis, 2011) and to support the survival and synaptic integrity of the auditory nerve (Mellado Lagarde et al., 2013). Damage to the organ of Corti is thought to result in loss of neurotrophin production and secondary degeneration of the spiral ganglion (Lawner et al., 1997; Staecker and Garnham, 2010). In a number of animal models, the delivery of various growth factors to the cochlea has resulted in increased preservation of neurons, re-growth of peripheral processes, and reduction of the threshold of electrical stimulation, suggesting that combining delivery of neurotrophins or neurotrophin mimetics may improve cochlear implant function (Evans et al., 2009; Wan et al., 2014; Budenz et al., 2015; Ramekers et al., 2015; Wise et al., 2016; Pfingst et al., 2017). Pathway analysis suggests that alterations in neurotrophin signaling should result in a broad range of physiologic changes in the cochlea. BDNF in particular appears to play an important role in both the peripheral and central auditory system (Singer et al., 2014). Despite the vast number of preclinical data concerning BDNF and cochlear health, nothing is known hitherto for the human auditory system. Interestingly, prior evaluations to the proteome of human perilymph (Schmitt et al., 2017) did not detect endogenous BDNF. The raw data of all detected proteins in perilymph by mass spectrometry in detail can be perused in the supplementary material of this prior study (Schmitt et al., 2017).

In order to determine whether BDNF and its regulated proteins are expressed in the adult human cochlea, we performed gene array analysis on human cochlear tissue. The results obtained from gene expression analysis were correlated with the previous reported proteome data of human perilymph samples (Schmitt et al., 2017). Based on the neurotrophin hypothesis, we assumed that patients without any RH would demonstrate altered neurotrophin signaling when compared to patients with RH and this would be reflected on the individual proteomic profiles of the perilymph. To evaluate this, we correlated the presence of BDNF-regulated proteins to preoperative hearing levels and postoperative cochlear implant performance data of the patients.

## Materials and Methods

### Perilymph and Cochlear Tissue Sampling

Collection of human cochlear tissue for gene array analysis was approved by the University of Kansas School of Medicine Institutional Review Board. Cochleae from three patients with normal hearing were obtained during a transcochlear approach to the posterior fossa for removal of meningioma and analyzed for growth factor gene expression.

Human perilymph was collected with a modified micro glass capillary during inner ear surgeries from 38 patients including three patients undergoing a bilateral cochlear implantation (37 perilymph samples during cochlear implantations and 4 during vestibular schwannoma surgeries) as already described in our previous study (Schmitt et al., 2017). The present study is an extension of a previous study (Schmitt et al., 2017). The data were analyzed with regard to pre-implantation hearing thresholds and cochlear implantation outcome. Patients aged 9 months up to 80 years with different etiologies for sensorineural hearing loss (e.g., MenieÌre’s disease, connatal cytomegalovirus infection, large vestibular aqueduct, CHARGE syndrome, meningitis, and auditory neuropathy) were included in this study. In addition, included patients were implanted with different cochlear implant devices and electrode arrays. A short version of the demographic data of the 38 patients undergoing cochlear implantation with perilymph sampling is depicted in Table 1. Detailed information about the individual patients is presented in the supporting information (Supplementary Table S1). The perilymph samples were obtained by puncturing of the round window membrane directly before the insertion of a cochlear implant electrode array. Protocols for collection of specimens were approved by the Ethics Committee of Hannover Medical School for perilymph by cochlear implantation (approval no. 1883-2013) and for perilymph during translabyrinthal vestibular schwannoma surgeries (approval no. 2403-2014).

**Table 1:** Demographic data of the 41 patients undergoing CI surgery with perilymph sampling.

Demographic data	Age (mean in years)	*n*^∗^ (%)
Patients	44.4	41 (100)
Male	44.3	23 (56.1)
Female	44.7	18 (43.9)
No hearing (NH)	36.5	27 (65.9)
Residual hearing (RH)	59.8	14 (34.1)
Children (0–18 years)	2.7	12 (29.3)
Adults (19–80)	61.7	29 (70.7)

*^∗^Bilateral samples of patients (n = 3) counted as two individual patients.*

Written informed consent was obtained from every patient included in this study.

### Genomic Evaluation

Tissue of the cochleae from three patients with normal hearing, undergoing a transcochlear approach to the posterior fossa for removal of meningioma were analyzed for growth factor gene expression. The lateral wall, basilar membrane and a portion of the modiolus were removed with an alligator forceps and immediately placed in RNAlater (Qiagen, cat #76104). Total RNA was extracted with Trizol reagent (Thermo Fisher Scientific, cat #15596018) and purified by centrifuging with phase lock heavy gel (Tiagen, cat # WMS-2302830). RNA was analyzed using the Agilent RNA6000 Pico kit in an Agilent Bioanalyzer 2100 to identify RNA degradation if present. Using an Affymetrix GeneChip WT Pico labeling kit, 50 ng of total RNA was TdT end labeled with biotin and hybridized on an Affymetrix HTA 2.0 array. After washing and staining, the chip was read with an Affymetrix GeneChip Scanner 3000 7G using a single scan with default normalization. Expression of genes present at 2 logs over baseline was evaluated. Data (genomic and proteomic) were uploaded into IPA software (Qiagen Bioinformatics) and evaluated based on the effects of varying expression of BDNF. Some proteins were subjected to classification by GOA using *UniProt* (UniProt Consortium, 2015).

### Proteomic Analysis

In a prior study, intraoperative perilymph sampling method and analysis by an in depth shot gun proteomics approach were established allowing the analysis of hundreds of proteins simultaneously in very small sample sizes in a microliters range (Schmitt et al., 2017). Perilymph samples were prepared for LC-MS/MS analysis by alkylation and separated using sodium dodecyl sulfate polyacrylamide gel electrophoresis as previously described (Schmitt et al., 2017). Peptide samples were separated with a nano-flow ultra-high pressure liquid chromatography system (RSLC, Thermo Fisher Scientific) equipped with a trapping column (3 μm C18 particle, 2 cm length, 75 μm ID, Acclaim PepMap, Thermo Fisher Scientific) and a 50 cm long separation column (2 μm C18 particle, 75 μm ID, Acclaim PepMap, Thermo Fisher Scientific). The RSLC system was coupled online via a Nano Spray Flex Ion Source II (Thermo Fisher Scientific) to an LTQ-Orbitrap Velos mass spectrometer. Metal-coated fused-silica emitters (SilicaTip, 10 μm i.d., New Objectives) and a voltage of 1.3 kV were used for the electrospray. Overview scans were acquired at a resolution of 60 k in a mass range of m/z 300–1600 in the orbitrap analyzer and stored in profile mode. The top 10 most intensive ions of charges two or three and a minimum intensity of 2000 counts were selected for CID fragmentation with a normalized collision energy of 38.0, an activation time of 10 ms and an activation Q of 0.250 in the LTQ. Fragment ion mass spectra were recorded in the LTQ at normal scan rate and stored as centroid m/z value and intensity pairs. Active exclusion was activated so that ions fragmented once were excluded from further fragmentation for 70 s within a mass window of 10 ppm of the specific m/z value. The relative protein quantification was performed by LFQ and was determined as LFQ intensity (Schmitt et al., 2017, 2018).

Additionally, proteins were subjected to classification by GOA using *UniProt* (UniProt Consortium, 2015). The Gene Ontology (GO) classification allows a mapping of the proteins into the categories *molecular function*, *biological process*, and *cellular compartment*. Proteins were described using a standardized vocabulary of the *UniProt* Knowledgebase by uploading the UniProt IDs of the proteins to the *UniProt* website http:/www.uniprot.org.

### Audiology: Classification of Patients by Audiogram Data

Before surgery and perilymph sampling, pure tone audiometry was performed using a calibrated audiometer according to DIN EN 60318 to detect the acoustic hearing threshold. The test method follows DIN ISO 8253 with headphones for air conduction and headset for bone conduction in both ears. The PTA was analyzed for the frequency region of 500, 1000, and 2000 Hz. This classification was used for a direct correlation of the hearing loss of patients with LFQ intensity of detected proteins.

For the classification of severity of hearing loss in decibels (dB) and percent hearing impairment, a previously described method was used (Shearer et al., 1993). Therefore, the PTA was calculated for the frequency region of 500, 1000, 2000, and 3000 Hz. To obtain an ear-specific level, 25 dB was subtracted from this PTA and multiplied by the factor 1.5. This method includes a broader range of frequency values and allows a more precise classification of severity of hearing loss, especially in our cochlear implant patients with severe hearing loss in the low frequency area. The preoperative audiograms were used to assign patients to one of the two groups:

No hearing: PTA threshold of 91 dB or higher, 100% hearing impairment, *n* = 27 ears.

Residual hearing: PTA threshold of less than 91 dB, some RH, *n* = 14 ears.

Additionally, the postoperative hearing performance with a cochlear implant 1 year after implantation of the 38 patients with perilymph sampling was analyzed. Therefore, data of three audiologic tests (HSM sentence test in quiet and in noise at 10 dB, Freiburg monosyllable word test) 1 year after implantation were included. From the 29 adult patients, the majority of the patients participated in the three tests: HSM sentence test in quiet (*n* = 15), HSM sentence test in noise 10 dB (*n* = 20), Freiburg monosyllable word test (*n* = 22). For children, these types of tests were not applicable.

**FIGURE 1:**
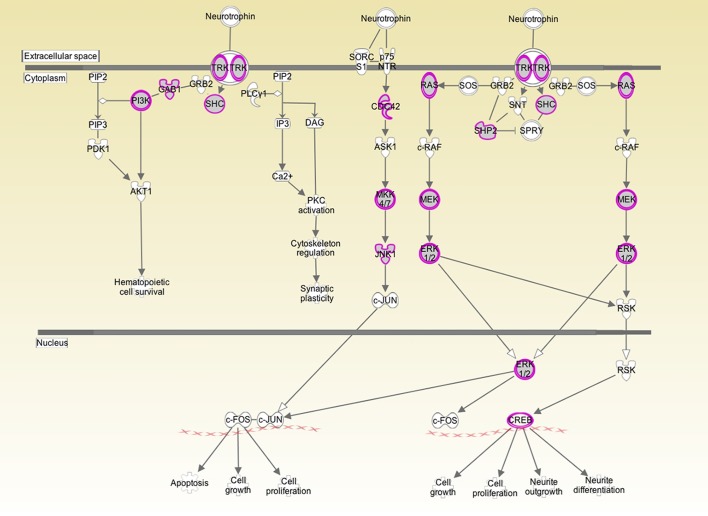
Analysis of human inner ear transcriptome over-layed on a generic human neurotrophin signaling pathway. Purple/gray proteins represent gene products/proteins identified at 10 log folds expression over baseline in the cochlear transcriptome. This suggests that those particular signaling pathways play an active role in the inner ear. Both the high affinity Trk receptor which is found in the spiral ganglion and the low affinity p75NTR which is thought to be in Schwann and glial cells are represented. p75NTR was detectable at 2 log fold expression over baseline expression. The downstream signaling genes were expressed at significantly higher levels.

### Statistical Analysis

Mass spectrometric raw data were processed using Max Quant software (version 1.4) and human entries of Swissprot/UniProt database. The threshold for protein identification was set to 0.01 on peptide and protein level.

Data were evaluated with multiple correlation analysis, or generation of heat maps and analysis with Fisher’s exact test (Graph Pad Prism V 7). Significance was set at *p* < 0.05.

## Results

### BDNF and TrkB Are Expressed in the Normal Hearing Human Cochlea

There is only very limited data on neurotrophin signaling in the adult human cochlea. Expression of BDNF mRNA and associated signaling molecules were determined by evaluating cochlear cDNA libraries from patients with normal hearing undergoing surgical removal of rare meningiomas anterior to the brainstem via a transcochlear approach. In these surgeries, hearing was sacrificed to gain better access to the tumor and thus also to the normal hearing cochlea.

Both BDNF and the TrkB receptor were expressed in all three specimens. The array did not differentiate between transcript variants. The low affinity p75NTR was also detectable in all three specimens. To determine the biologically significant pathways regulated by BDNF in the cochlea, we determined which components of neurotrophin signaling were present at a high level in the normal inner ear. As depicted in Figure 1, there is a high level of expression of the Ras/ERK pathway members and of CREB. These pathways are not only specific to neurotrophin signaling but may provide targets for pharmacological intervention for experimental or therapeutic purposes.

**Table 2:** Genes predicted to be regulated by BDNF in the normal cochlea.

Symbol	Entrez gene name	Location
*ATAT1*	Alpha tubulin acetyltransferase 1	Cytoplasm
*BDNF*	Brain derived neurotrophic factor	Extracellular space
*C5orf34*	Chromosome 5 open reading frame 34	Other
*CADPS2*	Calcium dependent secretion activator 2	Plasma membrane
*DNAJC21*	DnaJ heat shock protein family (Hsp40) member C21	Other
*HIST1H2AL*	Histone cluster 1H2A family member I	Nucleus
*ITIH3*	Inter-alpha-trypsin inhibitor heavy chain 3	Extracellular space
*MALAT1*	Metastasis associated lung adenocarcinoma transcript 1 (non-protein coding)	Nucleus
*MYH8*	Myosin heavy chain 8	Cytoplasm
*OMG*	Oligodendrocyte myelin glycoprotein	Plasma membrane
*PRSS12*	Protease, serine 12	Extracellular space
*RPL35A*	Ribosomal protein L35a	Cytoplasm
*SEMA3E*	Semaphorin 3E	Extracellular space
*SLC16A7*	Solute carrier family 16 member 7	Plasma membrane
*TMEM45A*	Transmembrane protein 45A	Plasma membrane

### BDNF Is Predicted to Regulate a Variety of Genes Involved in Neuronal Health in the Human Cochlea

Ingenuity Pathway Analysis software was used to identify pathways related to BDNF signaling that are expressed in all 3 cDNA samples from the normal hearing patients described above at 10-fold higher levels than baseline gene expression levels. In addition, the same software was used to identify the expression of genes in the normal cochlea that are predicted to be regulated by BDNF. A range of proteins, many of which have not been evaluated for their effect on hearing and cochlear biology, have been predicted by the IPA Knowledgebase (Table 2). Functions of these genes include microtubule assembly, synaptic vesicle trafficking, protein folding, DNA binding, transcriptional regulation, actin binding, axonogenesis, myelination, ribosomal function, and differentiation. Proteins corresponding to the genes from the cochlear cDNA library (results from mRNA analysis) that were predicted to be regulated by BDNF were not found in the perilymph samples analyzed by mass spectrometry.

### BDNF Is Predicted to Regulate a Variety of Proteins That Are Also Present in Human Cochlear Perilymph

The perilymph proteome in 41 perilymph samples of 38 patients undergoing inner ear surgeries with and without RH was evaluated, imported into IPA and queried for predicted relationships between BDNF and the identified proteins. Proteins found in the perilymph at significant concentrations were analyzed by IPA. Some of these proteins correspond to genes that are also predicted to be regulated by BDNF. Using this approach, we were able to indirectly identify genes and proteins relevant to the inner ear and regulated by BDNF. These perilymph proteins and their corresponding genes anticipated to be up and down regulated by reduced BDNF expression and proteins that are predicted to be regulated by reduced BDNF but with no information about the direction of regulation are summarized in Table 3. The proteins in Table 3 are ranked by the occurrence of the protein in perilymph samples of the patients within the three subgroups (downregulated, upregulated, with no information about regulation). Proteins were not detected in every perilymph sample. Proteins listed above in the subgroups have less abundance in the samples of the patients, meaning the proteins are detected in a low number of samples. However, the proteins at the bottom of the subgroup lists are detected in many samples of our patient cohort. Depicted in Supplementary Figure S1 are the proteins detected in % of the patients. In conclusion, these 41 proteins of the 878 previously determined proteins present in perilymph (Schmitt et al., 2017) were identified in the IPA Knowledgebase as being regulated by BDNF (Table 3). There was a wide range of expression profile for the different proteins in different patients with some proteins being expressed in only a few of patients and others being found in every patient, depicted in the supporting information (Supplementary Figure S1).

**Table 3:** Perilymph proteins and corresponding abbreviations (gene names) and Uniprot IDs anticipated to be up and down regulated and with no information about regulation by simulation that cochlear BDNF is reduced.

Downregulated	Symbol	Uniprot ID
Neurexin-1 beta	*NRXN1*	F8WB18
Cell division control protein 42 homolog	*CDC42*	P60953
Ubiquitin carboxyl-terminal hydrolase isozyme L1	*UCHL1*	D6RE83
Myelin proteolipid protein	*PLP1*	P60201
Myelin basic protein	*MBP*	E9PMR5
Ryanodine receptor 3	*RYR3*	Q15413
14-3-3 protein gamma	*YWHAG*	P61981
Ryanodine receptor 2	*RYR2*	H7BY35
Osteopontin	*SPP1*	P10451
Protein kinase C-binding protein NELL2	*NELL2*	F8VVB6
Heat shock protein HSP 90-alpha	*HSP90AA1*	P07900
Neural cell adhesion molecule 1	*NCAM1*	Q92823
Glial fibrillary acidic protein	*GFAP*	P14136
**Upregulated**		
Tripeptidyl-peptidase 1	*TPP1*	O14773
Laminin subunit gamma-1	*LAMC1*	P11047
Dihydropyrimidinase-related protein 2	*DPYSL2*	Q16555
Reelin	*RELN*	P78509
Ferritin light chain	*FTL*	P02792
Plexin-B2	*PLXNB2*	O15031
Agrin	*AGRN*	O00468
Annexin A5	*ANXA5*	P08758
Filamin-B	*FLNB*	O75369
Ribonuclease inhibitor	*RNH1*	P13489
Filamin-A	*FLNA*	Q5HY54
78 kDa glucose-regulated protein	*HSPA5*	P11021
Neutrophil gelatinase-associated lipocalin	*LCN2*	P80188
Annexin A2	*ANXA2*	P07355
Phospholipid transfer protein	*PLTP*	P55058
Vimentin	*VIM*	P08670
**No information about regulation**		
Gamma-secretase C-terminal fragment 59 (Amyloid beta A4 protein)	*APP*	E9PG40
Carboxypeptidase E	*CPE*	P16870
Chromogranin-A	*CHGA*	P10645
Glutamine synthetase	*GLUL*	P15104
Inter-alpha-trypsin inhibitor heavy chain H3	*ITIH3*	Q06033
Vasorin	*VASN*	Q6EMK4
Protein S100-A9	*S100A9*	P06702
Fibrinogen beta chain	*FGB*	P02675
Plasminogen	*PLG*	P00747
Actin, cytoplasmic 1	*ACTB*	P60709
Apolipoprotein E	*APOE*	P02649
Alpha-2-macroglobulin	*A2M*	P01023

For a more detailed view, GOA analysis was performed with the 41 perilymph proteins. These proteins were classified into the categories *biological process*, *molecular function*, and *cellular component*. The proteins were considered in the GO terms *cellular components*. Most proteins are intracellular proteins located in cell part (mainly intracellular organelle parts), organelle (mainly membrane-bounded organelle), and membrane (especially plasma membrane). But also proteins located in extracellular region part like extracellular exosomes were identified. A table with detailed information about proteins detected in >80% of patients or with direct regulation by BDNF mapped to the category *cellular components* by GOA is exemplarily shown in Supplementary Table S2. The main *molecular function* of the considered perilymph proteins depicts besides molecular function regulation and catalytic activity clearly binding with focus on protein and ion binding. The perilymph proteins are involved in manifold *biological processes* with focus on biological regulation. Many proteins are also participated in cellular, developmental and metabolic processes as well as in cellular component regulation, response to stimulus and localization.

### Severity of Hearing Loss Correlates to Overall Lower Expression of Perilymph Proteins Predicted to Be Regulated by BDNF

**FIGURE 2:**
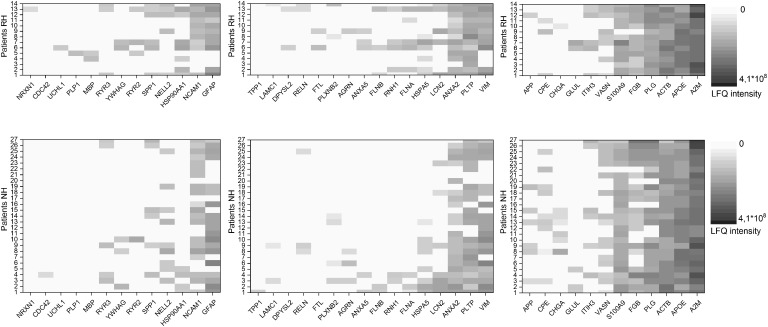
Heat map of proteins predicted to be regulated by BDNF. Shown are proteins anticipated to be downregulated with decrease in BDNF for patients with (RH) and without (NH) residual hearing on the left. Proteins anticipated to be upregulated with decrease in BDNF for patients with (RH) and without (NH) residual hearing in the middle and proteins with no information about the regulation with decrease in BDNF for patients with (RH) and without (NH) residual hearing on the right. Protein values of patients with RH are shown in the upper row, data of patients NH in the lower row. The concentration of the individual proteins was analyzed by label free quantification (LFQ) in human perilymph samples and an increase is shown with deeper shades of violet.

Using IPA software, we identified proteins detected in perilymph with direct or indirect regulation by BDNF and we grouped these regulated perilymph proteins by expected change in expression level when BDNF is decreased. The IPA Knowledgebase predicted that 13 proteins would be down regulated and 16 proteins would be up regulated when BDNF expression diminished (Table 3). No predictive information on up or down regulation could be found for the 12 remaining proteins (Table 3). Patients were divided into two groups, patients with and without RH based on a cutoff of 90 dB PTA, and correlated to the three groups of proteins regulated by BDNF. These results are shown as a heat map depicting the presence and concentration of the various proteins in patients with and without RH (Figure 2). Patients with no preoperative RH had significantly lower levels of the proteins predicted to be down regulated when BDNF decreases (Figure 2; left side; *p* = 0.007; Fisher’s exact test). For proteins that are predicted to be up regulated, there was also a significant difference between the groups (Figure 2; middle; *p* = 0.02; Fisher’s exact test). For proteins determined to be regulated by BDNF expression but without predictable change direction, there was no statistically significant difference between RH and non-hearing patients (Figure 2; right side).

### Preoperative Hearing Loss Correlates to Decreased Perilymph Expression of Phospholipid Transfer Protein

**FIGURE 3:**
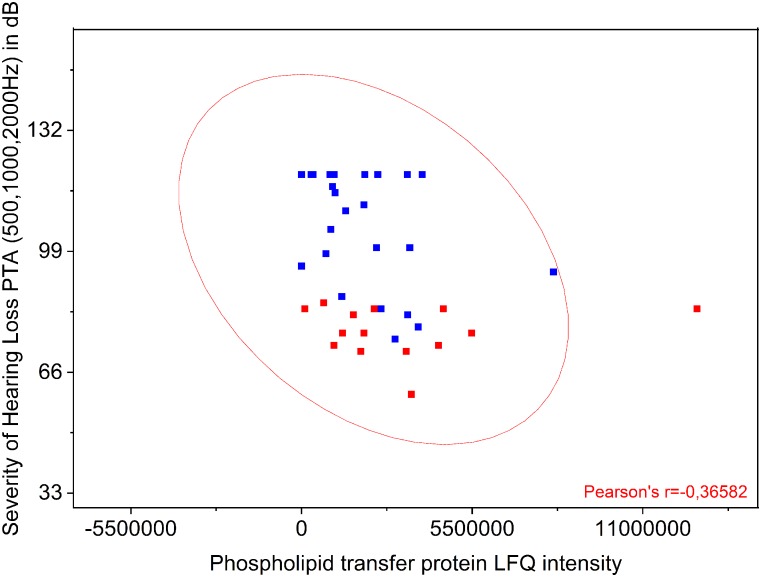
Comparison of PLTP protein levels to the patients’ preoperative pure tone average (PTA). Shown is a significant correlation between preoperative PTA (500, 1000, and 2000 Hz) and presence of PLTP. The protein levels were analyzed by label free quantification (LFQ intensity) in human perilymph samples.

The proteins listed in Table 3 were correlated to the preoperative hearing level and postoperative performance data with the cochlear implant 1 year after implantation. Comparison of protein levels (LFQ intensity) to the patients’ preoperative PTA showed a slightly significant correlation between preoperative PTA and level of phospholipid transfer protein (PLTP) (Pearson *r* = −0.3658; *p* = 0.0187) shown in Figure 3. Correlation of the other proteins to the PTA was not significant. PLTP was detectable in the perilymph of the majority of the patients (36/41).

**FIGURE 4:**
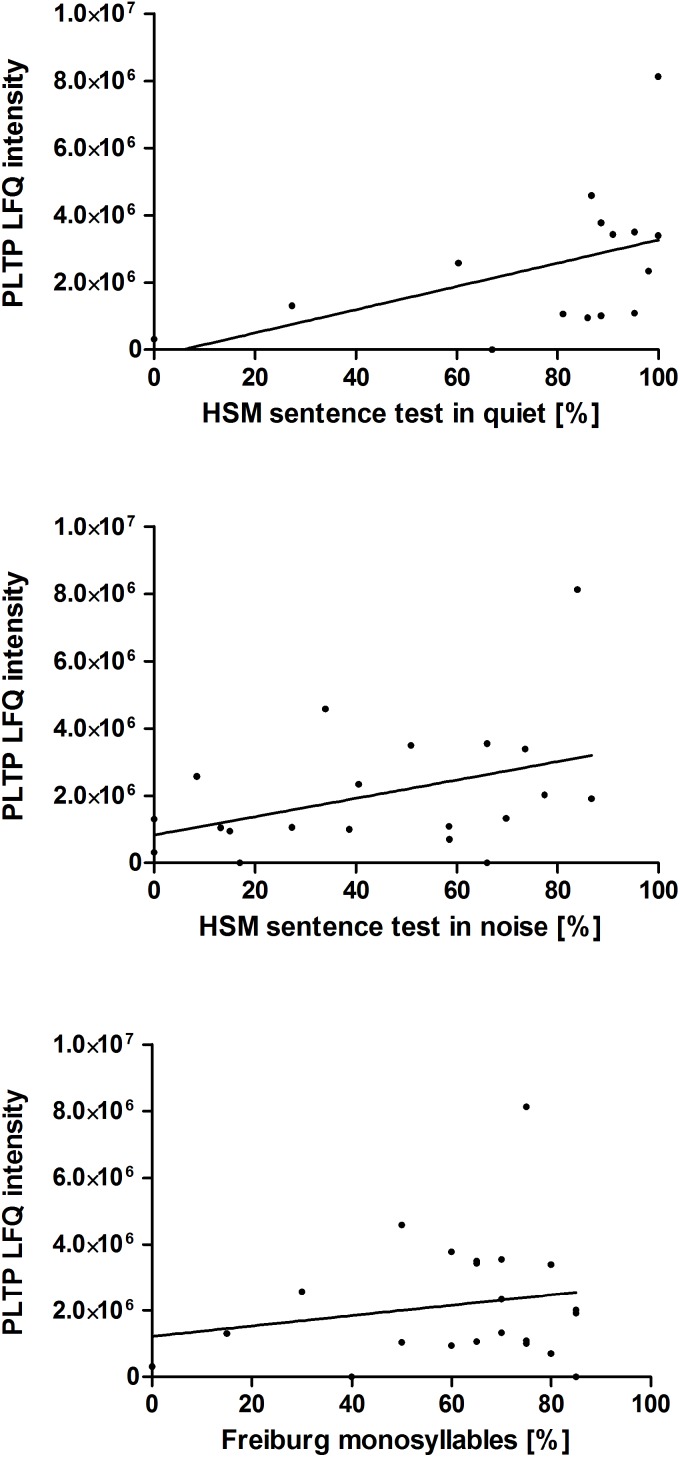
Correlation of the PLTP protein levels to the patients’ postoperative hearing performance. Audiologic test data of 29 adult patients of the 41 patients with perilymph sampling 1 year after cochlear implantation are depicted as speech understanding in %. The HSM sentence test in quiet (*n* = 15) is shown on the top, the HSM sentence test in noise at 10 dB (*n* = 20) in the middle and the Freiburg monosyllable word test (*n* = 22) at the bottom. The protein levels were analyzed by label free quantification (LFQ intensity) in human perilymph samples.

Additionally, the correlation of the relative PLTP concentration (LFQ intensity) in perilymph at the time point during surgery to postoperative hearing performance 1 year after hearing restoration with a cochlear implant was analyzed. Data of audiologic tests (HSM sentence test in quiet and in noise 10 dB, Freiburg monosyllable word test) 1 year after implantation were used for a correlation analysis. Data of the majority of the patients, children excluded, were analyzed: HSM sentence test in quiet (*n* = 15), HSM sentence test in noise 10 dB (*n* = 20), Freiburg monosyllable word test (*n* = 22). There might be a trend toward better understanding in patients with higher PLTP levels in perilymph is shown in Figure 4. For confirming these hypotheses further studies are required.

## Discussion

The integrity of the spiral ganglion is thought to play a key role in cochlear implant function. Since the studies of Spoendlin demonstrated that aminoglycoside injury of the peripheral auditory system in cats resulted in a secondary degeneration of the spiral ganglion (Spoendlin, 1975), there has been significant interest in understanding the mechanisms that maintain spiral ganglion integrity and function. In humans, the process of spiral ganglion degeneration appears to occur at a different rate than in animal models. There clearly are variations in spiral ganglion populations that occur with different disease processes (Otte et al., 1978). The relationship between spiral ganglion integrity and cochlear implant outcomes in humans has been more difficult to establish. Initial temporal bone studies did not show a clear relationship between spiral ganglion counts and implant outcomes (Fayad et al., 1991). More recent studies with larger numbers, however, do show a relationship between speech outcomes and the quantity of spiral ganglion neurons (Seyyedi et al., 2014). To assess the functionality of the remaining spiral ganglion neurons, animal studies have been used to correlate changes in the electrically evoked compound actual potential of guinea pigs with intact or impaired spiral ganglion neurons (Pfingst et al., 2015b). This potentially correlates to measurable changes in patients undergoing cochlear implantation since an abnormal growth function of the electrically evoked compound action has been correlated to poor cochlear implant speech outcomes (Kim et al., 2010).

An important question that arises is whether neurotrophic factors can influence neuronal survival in humans treated with cochlear implants. Amongst the most studied candidates are the neurotrophins. The function of BDNF in the adult rodent auditory system was recently reviewed (Singer et al., 2014). Diverse studies mainly using knockout and conditional knockout mice suggest that BDNF is initially expressed in auditory hair cells. During maturation of hearing, the pattern of BDNF expression changes to inner phalangeal cells, pillar cells (the cells surrounding the inner hair cell) and spiral ganglion cells (Fritzsch et al., 2004; Kersigo and Fritzsch, 2015; Johnson Chacko et al., 2017). One of the key roles of BDNF in the adult auditory system may include maintenance of ribbon synapse integrity (Zuccotti et al., 2012; Singer et al., 2014). The role of BDNF in the human adult auditory system is less defined. A number of studies have looked at the expression of neurotrophin and their receptors in normal adult human cochlear tissue from cochleae harvested during acoustic neuroma surgery. The TrkB receptor was found to be expressed in the spiral ganglion neurons and in nerve fibers underneath outer hair cells (Liu et al., 2011). BDNF expression was not detectable by immunohistochemistry (Liu et al., 2011). A recent *in situ* hybridization study in adult human inner ear tissue, however, has found BDNF mRNA in auditory hair cells and supporting cells (Johnson Chacko et al., 2017). Expression of the low affinity p75NTR was demonstrated in Schwann cells and glial cells but not in spiral ganglion neurons (Liu et al., 2012). Currently, no human data exists demonstrating what changes in neurotrophin signaling occur as a result of hearing loss. The signaling pathways of BDNF are well understood. Consequently, a potential method of evaluating BDNF effects in the human inner ear is to look at the downstream effects of BDNF signaling.

In the central nervous system, there are clear links to a variety of neurodegenerative pathologies and decreased levels of BDNF expression (Lu et al., 2013). Besides direct degeneration of the tissues producing BDNF, the expression of BDNF can be modulated by inflammatory mediators which may be present as a consequence of cochlear implantation (Calabrese et al., 2014). Additionally, as shown in many studies, BDNF can autoregulate its expression (Zheng et al., 2012). Animal studies have shown that implant function can be improved after cochlear damage by either co-infusing BDNF or inducing production of BDNF through gene therapy (Staecker and Garnham, 2010; Budenz et al., 2012). A key factor in translating these finding into human studies is to identify patients with insufficient endogenous BDNF production or signaling. In addition to electrophysiological measures (Pfingst et al., 2015a), direct or indirect indicators of neurotrophin function in perilymph may be detectable. Unlike in CSF (Calderon-Garciduenas et al., 2016; Li et al., 2016; Martin-de-Pablos et al., 2018), direct detection of BDNF by mass spectrometry have not been demonstrated in perilymph yet (Schmitt et al., 2017).

In the herein presented study, analysis of gene expression in three normal human patients showed the presence of BDNF, TrkB and the p75NTR in the cochlea, supplementing information gained from prior immunohistochemical studies. We compared these data with analysis of proteins in the perilymph of patients undergoing cochlear implantation, therefore having hearing loss, and analyzed their relationship to BDNF signaling. Based on profiling, we show that expression of signaling cascades downstream from neurotrophic tyrosine kinase receptors and the low affinity p75NTR are active in the normal cochlea (Figure 1). There is active expression of PI3K, CDC42, and RAS (Figure 1) resulting in activation of pathways that maintain cell survival, growth, and neuritogenesis (Sandhya et al., 2013). Using IPA software, we identified a subgroup of the 878 expressed perilymph proteins, identified, and described in a previous study (Schmitt et al., 2017), that may be regulated by BDNF (Table 3). The 41 proteins identified based on their relationship to BDNF signaling may be related to the hearing level of the patients since different protein levels between the two patients groups were analyzed (patients with RH and patients with profound hearing loss).

For the proteins that are predicted to be down regulated if BDNF is reduced, patients with no RH prior to implantation expressed lower or absent levels of these proteins. For this group of proteins, ryanodine receptors 2 and 3, and HSP 90 are hair cell specific, so their reduction could be a reflection of loss of hair cells rather than changes in BDNF signaling (Lim et al., 1996; Mammano et al., 2007). Within this group, NELL2 is a spiral ganglion marker (Nelson et al., 2002). GFAP is a marker for spiral ganglion glial cells and supporting cells (Rio et al., 2002) and NCAM1 is associated with the spiral ganglion (Terkelsen et al., 1989). Individually, these proteins did not correlate to preoperative hearing levels. A potential explanation of this is found in the adult rodent data. If BDNF really is only expressed in the higher frequency region of the adult cochlea as suggested by Zuccotti et al. (2012), then our RH and non-hearing patients may not have a significant difference in BDNF expression since most patients undergoing implantation have RH only at lower frequencies. A larger cohort of patients with variable hearing loss will be needed to further refine these outcomes. Interestingly, among these proteins osteopontin is a marker for vestibular hair cells and may also be expressed in lower levels in the marginal cells and in the spiral ganglion (Sakagami, 2000). This protein has a decreased expression in patients without RH, suggesting that there may also be differences in balance function that can be assayed through this approach.

For the proteins that were predicted to be upregulated when BDNF is reduced, filamin A and B are actin binding proteins which can be found in hair cell stereocilia (Ramakrishnan et al., 2012). Plexins are members of a family of transmembrane proteins that control axonal growth and have been demonstrated in the developing spiral ganglion (Lu et al., 2011). Annexin A5 has been described as being present in high concentration in hair cell stereocilia (Krey et al., 2016). Within this group, the patients with profound hearing loss expressed a significantly reduced proteome, when considering proteins regulated by BDNF (Figure 2). Again, this may reflect the overall degeneration of the organ of Corti rather than a BDNF regulatory effect. Within this subgroup, however, phospholipid transfer protein showed a statistically significant correlation to preoperative PTA. This protein is generally identified with lipid metabolism but has been identified in the developing inner ear as expressed in cells of a non-hair cell lineage during development (Scheffer et al., 2015). By GOA the molecular, function of this protein is characterized by lipid transporter activity (including phospholipids and ceramides like diacylglycerol, phosphatidic acid, sphingomyelin, phosphatidylcholine, phosphatidylglycerol, cerebroside, and phosphatidyl ethanolamine). Additionally, this protein is involved in different biological processes like ceramide transport, flagellated sperm motility, high-density lipoprotein particle remodeling, lipid metabolic process, lipid transport, phospholipid transport, positive regulation of cholesterol efflux, and vitamin E biosynthetic process. Interestingly, a link between PLTP and the amyloid precursor protein (APP) was discussed suggesting that a deficiency of these proteins accelerates memory dysfunction in mouse models. Additionally, PLTP might play an important role in autophagy modulation (Tong et al., 2015). Its function in the adult inner ear is not defined yet.

When discussing the results obtained from the present study, several limitations need to be considered. The population of the patients included into our analysis is highly heterogenous with different anatomic and molecular pathologies. This can certainly add to the variability in the perilymph proteome that we showed in this study and in prior studies (Schmitt et al., 2017, 2018). In addition, different cochlear implant devices with various electrode arrays have been used for the treatment of the hearing loss, adding more variables to be considered when analyzing the hearing performance with the device. Finally, the low number of patients included and the vast number of proteins identified in the perilymph are limiting the statistical power of the study. The influence of age and gender on gene and protein expression needs also consideration. Thus, future studies need to concentrate on a higher number of homogenous groups of patients.

Despite the limitations, evaluation of the perilymph proteome offers a novel approach for the indirect evaluation of a wide range of biological effects within the cochlea of patients undergoing cochlear implantation. Animal studies have demonstrated that BDNF supplementation can enhance spiral ganglion survival (Evans et al., 2009; Wan et al., 2014; Budenz et al., 2015; Ramekers et al., 2015; Wise et al., 2016; Pfingst et al., 2017). Despite temporal bone pathology and physiological evidence that spiral ganglion survival is variable between disease processes and affects cochlear implant outcomes, we have a very incomplete understanding of the diseased human inner ear. Cochlear “health” is hypothesized to have a significant impact on cochlear implant outcomes (Pfingst et al., 2015b, 2017). The human perilymph proteome in implant patients consists of greater than 800 individual proteins. By focusing on proteins that are regulated by BDNF, we can analyze subgroups that may reflect changes in BDNF signaling or even allow the identification of markers that may reflect BDNF signaling or demonstrate degeneration of hair cells and supporting cells within the organ of Corti.

## Conclusion

We have demonstrated that BDNF is expressed in cochlear tissue in normal hearing individuals. Patients with profound hearing loss have less BDNF-regulated proteins in their perilymph when compared to patients with some RH. In addition, the expression level of PLTP correlated not only to preoperative hearing but also tend to improved outcome with cochlear implants in individual patients. This initial study identifies a group of proteins that may potentially serve as markers for inner ear health as it relates to cochlear implantation. In order to use this tool for the identification of biomarkers or for the reliable prediction of cochlear implant outcome, large applied studies need to be performed.

## Data Availability

The datasets analyzed for this study can be found in the Gene Expression Omnibus (GEO, accession number GSE128505).

## Author Contributions

IdV acquired and analyzed the data, and wrote the first draft of the manuscript. HS processed and analyzed the perilymph, and wrote the manuscript. TL designed the study, provided financial support, and approved the manuscript. NP collected the perilymph, provided and analyzed the human data, and approved the manuscript. SA performed the bioinformatics analysis of the data, and approved the manuscript. HS designed the study, performed the bioinformatics analysis of the data, and wrote the manuscript. MD collected the perilymph, provided and analyzed the human data, approved the manuscript, and provided financial support. AW designed the study, collected the perilymph, analyzed the data, and wrote the manuscript.

## Conflict of Interest Statement

The authors declare that the research was conducted in the absence of any commercial or financial relationships that could be construed as a potential conflict of interest.

## Abbreviations

BDNF: brain derived neurotrophic factor
CDC42: cell division control protein 42 homolog
CID: collision-induced dissociation
CSF: cerebrospinal fluid
GFAP: glial fibrillary acidic protein
GOA: gene ontology annotations
HSP 90: heat shock protein 90
HTA: human transcriptome array
ID: inner diameter
IPA: Ingenuity Pathway Analysis
IRB: Institutional Review Board
LTQ: linear trap quadrupole
NCAM1: neural cell adhesion molecule 1
NELL2: neural tissue-specific epidermal growth factor-like repeat domain-containing protein
LFQ: label free quantification
NH: no hearing
NT-3: neurotrophin 3
p75NTR: p75 neurotrophin receptor
PI3K: phosphoinositide 3-kinase
PTA: pure tone average
RAS: rat sarcoma
RH: residual hearing
TdT: terminal deoxynucleotidyltransferase
Trk: receptor tyrosine kinase
