# Supplementary material for: Detection of BDNF-Related Proteins in Human Perilymph in Patients With Hearing Loss
Source: Front Neurosci. 2019 Mar 26;13:214. doi: 10.3389/fnins.2019.00214 (PMC6445295; doi:10.3389/fnins.2019.00214)

Supplementary material

Table S1: Table of detailed demographic and etiologic data of patients with inner ear surgeries and perilymph sampling; XLSX

Table S2: The 9 proteins expressed in >80% of the patients or proteins directly related to the BDNF pathway were mapped to the category *cellular components* for detailed GOA profiles by Uniprot.

Gene Name	Entrez Gene Name	Cellular Component
PLTP	Phospholipid transfer protein	extracellular region part<br>extracellular region<br>protein-containing complex
APP	Gamma-secretase C-terminal fragment 59 (Amyloid beta A4 protein)	extracellular region part<br>organelle<br>organelle part<br>cell part<br>extracelular region<br>membrane<br>organelle lumen<br>protein-containing complex<br>membrane part<br>cell junction<br>synapse part<br>neruomuscular junction
CPE	Carboxypeptidase E	extracellular region part<br>organelle<br>organelle part<br>cell part<br>membrane<br>membrane part<br>synapse part
S100A9	Protein S100-A9	extracellular region part<br>organelle<br>organelle part<br>cell part<br>extracellular region<br>membrane<br>organelle lumen<br>cell junction
FGB	Fibrinogen beta chain	extracellular region part<br>organelle<br>organelle part<br>cell part<br>extracellular region<br>membrane<br>organelle lumen<br>protein-containing complex<br>membrane part
PLG	Plasminogen	extracellular region part<br>organelle<br>organelle part<br>cell part<br>extracellular region<br>membrane<br>organelle lumen<br>other organism cell<br>membrane
ACTB	Actin, cytoplasmic 1	extracellular region part<br>organelle

		organelle part<br>cell part<br>membrane<br>protein-containing complex<br>cell junction
APOE	Apolipoprotein E	extracellular region part<br>organelle<br>organelle part<br>cell part<br>extracellular region<br>membrane<br>organelle lumen<br>protein-containing complex
A2M	Alpha-2-macroglobulin	extracellular region part<br>organelle<br>organelle part<br>cell part<br>extracellular region<br>organelle lumen

Figure S1: Presence of proteins involved in BDNF-regulation detected in perilymph samples of patients with (RH) and without (NH) residual hearing. Proteins were not detected in every perilymph sample. Depicted here are the proteins detected in % of the perilymph samples. Shown are proteins anticipated to be downregulated by BDNF decrease in the left group, anticipated to be upregulated by BDNF decrease in the middle and with no information about the direction of regulation in the right group. NH (n=27), RH (n=14)

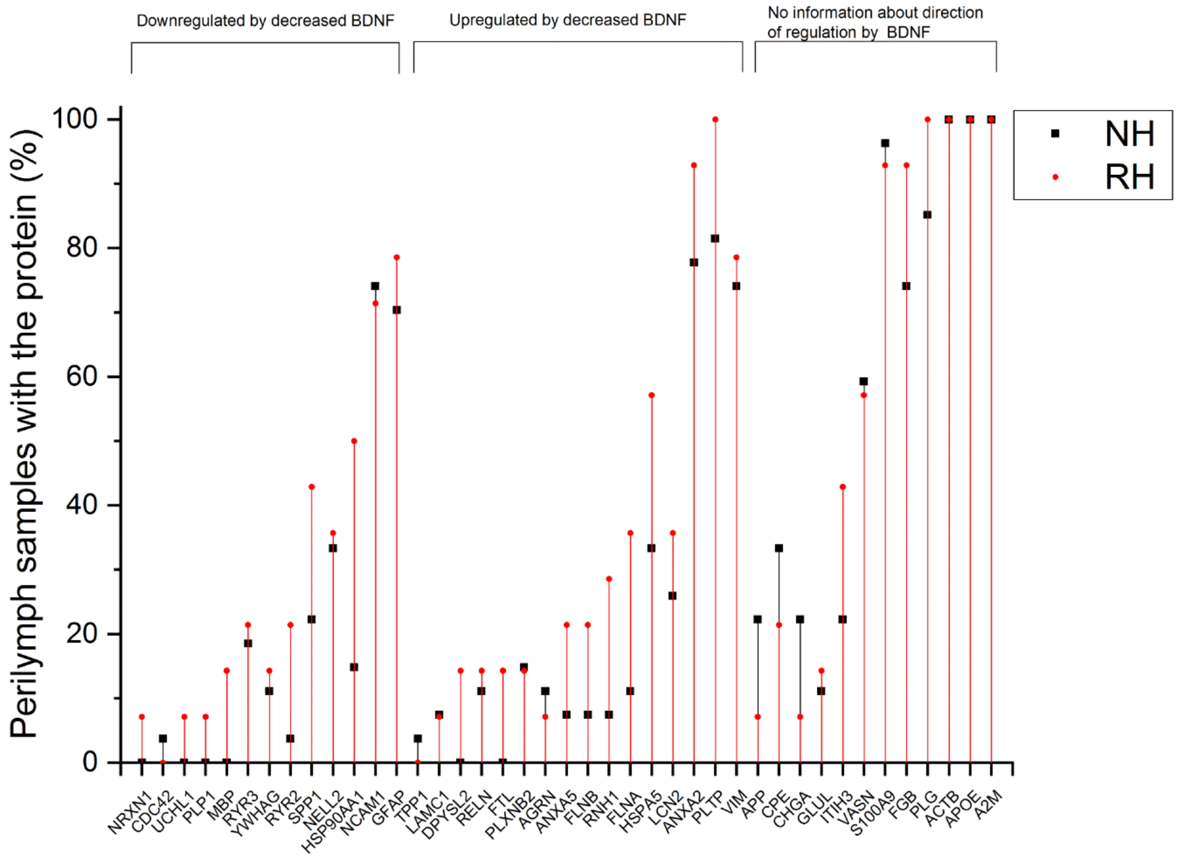

Supplement: Supplementary file 2 [file Data_Sheet_1.PDF]
